# Recent changes in women’s Olympic shooting and effects in performance

**DOI:** 10.1371/journal.pone.0216390

**Published:** 2019-05-13

**Authors:** Daniel Mon-López, Carlos M. Tejero-González, Santiago Calero

**Affiliations:** 1 Physical Education, Sport and Human Movement Department, Autonomous University of Madrid. Spain; 2 Universidad de las Fuerzas Armadas ESPE. Sangolquí. Ecuador; Universita degli Studi di Verona, ITALY

## Abstract

In 2018, the Olympic shooting regulations were modified to increase the number of women’s shots from 40 to 60, equaling the number given to men. This research presented in this paper addresses two research issues: (1) has the performance of women’s shooting changed as a result of this increase in the number of shots? and (2) with the equalized number of shots in place, do women and men perform differently? This study included 292 shooters who competed in the 2016 and/or 2018 European Championships who all obtained top-50 results. Our sample included balanced quotas for sports (50% pistol and 50% rifle) and by category (50% women and 50% men). Both championships were held in the same facilities and in the same month of the season, but with the difference that in 2016, women had 40 shots and in 2018 they had 60 shots. We observed that women’s performances did not diminish for the pistol or the rifle category when their number of shots were increased. Men and women shot equally well with rifles, although the men’s performance with pistols was higher than that of women. We concluded that sports in which physical strength is a minor factor, as in the case of shooting, should revise their regulations in the interest of greater gender equality in sports.

## Introduction

Effective equality between men and women is a social and political goal and is an international legal principle recognized in the Declaration of Human Rights. Gender gaps still clearly exist in the world of sport [1], even though gender equality in terms of participation, the number of available sporting events, salaries, and performance have been the subject of extensive discussion and debate. In fact, because of the disparity in the Olympic Games program, the International Olympic Committee (IOC) Executive Board is promoting effective equality between men and women by approving 25 measures in different areas, including nine for International Federations. The IOC’s strategic objective around gender equality calls for growing the number of female sporting events and increasing participation at the Olympic Games to 50 per cent [2].

As part of this effort, the International Shooting Sport Federation (ISSF) made two important changes in 2018. First, the technical rules for new mixed team events were created in order to implement the IOC’s decisions regarding the Olympic shooting program for Tokyo 2020. Second, women and men were given equal numbers of shots. From January 1, 2018, all shooting events had the same numbers of shots for men and women with the new rules, the number of shots increased from 40 to 60 for women’s 10 m air rifle and air pistol events [3]. Thus, men and women now compete in the pistol and air rifle modalities with the same number of shots, facilitating (from a methodological point of view) comparisons of their performance [4]. In addition, these new regulations allow competition as mixed teams for the first time in Olympic shooting events [5]. These new Olympic shooting rules allow the performance between men and women to be approached in absolute terms and in so, increase the competition time which could result in changes in the average time required per shot.

Past research has indicated that women fatigue more than men when firing with certain types of rifles [6] which is related with a decrease in shooting performance in long and short tasks [7,8] as a result of reduced body stability among women [9]. More generally, in absolute terms, the performance of men in most sports exceeds that of women, and gender has been identified as a major determinant of this difference. This occurs through the impact of genetic and hormonal differences which exert themselves in terms of height, weight, body fat, muscle mass, aerobic capacity, and anaerobic threshold. For example, in athletes and swimmers, this can mean a difference in elite performances of around 10% [10]. Other researchers have found that in the world’s best athletes, the performance differences between the sexes are between 8% and 12% for most sporting events [11]. However, because of its unique demands, the gender divide in Olympic shooting may be different.

In the words of Goldschmied et al., “shooting is one sport where the physical demands placed on the athletes are relatively minor” [4]. However, several studies indicate that there is a relationship between certain skills and performance in this sport. For example, Mondal et al. suggested that “having general physical skill areas contributes to the firmness that will support shooting cardio-vascular, respiratory endurance, stamina, strength, flexibility, power, coordination, agility, balance and accuracy” [12]. In the same way, some authors relate the importance of motor skills such as balance, especially in women’s performance in pistol shooting [13], or of specific grip strength in the case of men [14], which, in Olympic-level shooting, could be interpreted as men and women using different shooting skills and techniques to attain similar levels of performance. In general, there seems to be a consensus that balance and shooting strength specific to the sport are the most important abilities in precision shooting [15].

However, questions remain about the physical abilities involved in shooting. For example, a study by Anderson et al. [16] showed that men had more strength and balance compared to women, and especially pointed to strength as a factor related to the higher levels of fatigue felt by women. Similar results were found in the study by Mondal et al. [12] in which male rifle shooters were stronger than women. Mon [13] also found that men had more grip, higher shoulder abductor muscle strength, and fewer center of pressure (COP) displacements in air pistol shooting compared to women. Another study looking at police pistol training also found that men have higher grip strength compared to women [17]. Going beyond these physical qualities, rifle shooting performance may also be influenced by fine motor coordination qualities, such as “holding ability, aiming accuracy, cleanness of triggering and timing of triggering” [18]. Similarly, psychological factors like anxiety can influence shooting performance and as such, novice shooters perform significantly worse as a result of elevated anxiety levels [19].

Nonetheless, it is important to note that some studies have not shown any differences in the performance between men and women in the Olympic air rifle or 22-caliber modalities in real competition, suggesting that these physiological differences may not influence performance [4]. Similarly, Kemnitz et al. [20] did not find any sex-related differences in military rifle shooting performance. In pistol shooting, research findings are also contradictory. For example, Mon [13] did not find any differences in the average points obtained per shot by men and women (where men and women were given 60 and 40 pistol shots, respectively). In contrast, Anderson et al. [16] found that male police officers performed better with pistols than female police officers. Similar results were found by Copay et al. [17], who observed that men performed better with 9 mm, 0.40 inch, and 0.45 inch caliber pistols than women. However, Vučković et al. [21] found differences in pistol performance at the beginning of a police academy training program, which disappeared by the end of the training period.

While many studies have explored gender performance in shooting, the results have often been conflicting. Some show that men perform better than women, while others indicate that performance is equal between the sexes, suggesting that this topic should be further explored. Thus, the recent change in the Olympic shooting rules to equalize the number of shots that men and women are allowed is a useful opportunity to study two specific objectives in this context: (1) has increasing the number of shots affected women’s performance? and (2) have the rule changes and equality in competition led to a gender gap in performance?

## Materials and methods

### Procedures

The data in this study were obtained from the public database of the International Shooting Sport Federation [22,23] for participants in the 2016 and 2018 European Championships. Both championships were held in the same facilities in the city of Gyor (Hungary) in the same month of the shooting season (February 22–28, 2016 and February 19–26, 2018). The study was primarily carried out after the 2018 Championships in which the new rules established by the ISSF were implemented to increase the number of women’s shots to 60, equal to men [3]. This study was approved by the Ethics Committee at the Autonomous University of Madrid (Spain).

### Participants

The sample included 292 shooters from 47 countries in 2016 and 48 countries in 2018, who participated in the 2016 and/or 2018 European Championships. All of them obtained top-50 results, and we established samples that were balanced in terms of sports (50% pistol and 50% rifle) and category (50% women and 50% men). We distinguished three groups for each competition and category: shooters who participated in the 2016 championship, shooters who participated in the 2018 championship, and shooters who participated in both championships. The configuration of the sample is detailed in Table 1.

**Table 1:** Sample descriptive data.

Event	European Championship	Category				*N*
		Women		Men		
		*n*	age	*n*	age	
Pistol 50%	2016	25	32	21	31	146
	2018	25	30	21	31	
	2016 and 2018	25	31^A^	29	33^A^	
Rifle 50%	2016	27	30	25	31	146
	2018	28	26	25	27	
	2016 and 2018	16	28^A^	25	30^A^	
		146		146		292

Abbreviations: *n* or *N* = group size. Notations: age = average age in years (without decimals), ^A^ = age in 2018.

### Variables

The following independent variables were analyzed: championship (2016 and 2018), category (women and men) and event (air pistol and air rifle). We used sports performance, calculated as the average of all the shots (the APS or Average Points Per Shoot variable) as a dependent variable because women fired 40 shots (4 sets of 10 shots) and men fired 60 (6 sets of 10 shots) in the 2016 championship, but in the 2018 championship both women and men were allowed 60 shots.

### Data analysis

We calculated descriptive statistics, reporting the arithmetic mean (*M*), standard deviation (*SD*), and the normality of the distributions using the Shapiro–Wilk statistic (*SW*), and inferential statistics using independent and paired *t*-tests (for independent and related samples, respectively), establishing a confidence level of 95% (*p* < 0.050). The size of the effect was estimated for statistically significant differences using, on the one hand, the delta parameter (*d*) and establishing three cut-off points: low effect (0.200), medium effect (0.500), and large effect (0.800); and on the other hand, using the difference in performance in terms of percentage ($\%\;=\;\frac{M1}{M2}\;\times 100)$. These calculations were carried out using the IBM SPSS Statistical package (version 25) and GPower 3.1 software.

## Results

The results of the shooters who participated in a single championship, either in 2016 or 2018, are detailed in Table 2. Starting with the independent data for the air pistol event, the scores of the women who participated in 2016 (*M* = 9.364) were statistically equal to the 2018 scores for women (*M* = 9.374) (*p* = 0.703). In addition, the men’s scores in 2016 (*M* = 9.541) were significantly higher than the women’s results, both in 2016 (*M* = 9.364) with a large effect size (*p* < 0.001, *d* = 2.060, *%* = 1.890) and in 2018 (*M* = 9.549) (*p* < 0.001, *d* = 2.270, *%* = 1.870). In relation to the air rifle event, the women’s scores in 2016 (*M* = 10.347) were significantly lower than the 2018 scores (*M* = 10.382) with an average effect size (*p* = 0.002; *d* = 0.790, *%* = 0.340). However, no significant differences were observed between men and women for air rifle performance either in 2016 (*M* = 10.347 vs. *M* = 10.343, *p* = 0.801) or 2018 (*M* = 10.382 vs. *M* = 10.379, *p* = 0.839).

**Table 2:** Results of the shooters who participated in a single championship. Data for independent *t*-tests.

Event and Category	European Championship							
	2016 Number of shots: 40 for women and 60 for men				2018 Number of shots: 60 for women and 60 for men			
	*n*	*M*	*SD*	*SW*	*n*	*M*	*SD*	*SW*
**Pistol**								
Women	25	9.364	0.111	0.055	25	9.374	0.067	0.105
Men	21	9.541	0.049	0.435	21	9.549	0.086	0.029
**Rifle**								
Women	27	10.347	0.048	0.064	28	10.382	0.028	0.018
Men	25	10.343	0.041	0.098	25	10.379	0.055	0.019

Abbreviations: *n* = group size, *M* = arithmetic mean, *SD* = standard deviation, *SW* = Shapiro–Wilk normality test.

The results of the shooters who participated in both the 2016 and 2018 championships are detailed in Table 3. Looking at the dependent data for the air pistol event, the women’s scores in 2016 (*M* = 9.425) were significantly lower than their 2018 scores (*M* = 9.473) with an average effect size (*p* = 0.020, *d* = 0.530, *%* = 0.110); while men’s scores in 2016 (*M* = 9.586) were significantly higher than the women’s results, both in 2016 (*M* = 9.425) with a large effect size (*p* < 0.001, *d* = 1.790, *%* = 1.710) and in 2018 (*M* = 9.563) (*p* = 0.001, *d* = 0.970, *%* = 0.900). Regarding the dependent data from the air rifle event, women’s scores in 2016 (*M* = 10.357) were statistically equal to their 2018 scores (*M* = 10.382) (*p* = 0.164). Men’s scores in 2016 (*M* = 10.387) were higher than those of women in 2016 (*M* = 10.357) with average effect size (*p* = 0.030, *d* = 0.690, *%* = 0.290), but in 2018, men’s scores (*M* = 10.399) were statistically equal to the women’s scores in the same event (*M* = 10.382) (*p* = 0.305).

**Table 3:** Results of the shooters who participated in both championships. Data for paired *t*-tests.

Event and Category	*n*	European Championship					
		2016 Number of shots: 40 for women and 60 for men			2018 Number of shots: 60 for women and 60 for men		
		*M*	*SD*	*SW*	*M*	*SD*	*SW*
**Pistol**							
Women	25	9.425	0.106	0.133	9.473	0.110	0.509
Men	29	9.586	0.070	0.202	9.563	0.071	0.046
**Rifle**							
Women	16	10.357	0.047	0.274	10.382	0.046	0.208
Men	25	10.387	0.039	0.391	10.399	0.056	0.294

Abbreviations: *n* = group size, *M* = arithmetic mean, *SD* = standard deviation, *SW* = Shapiro–Wilk normality test.

## Discussion

The main finding of this study is that women’s pistol and rifle shooting performance did not decline in either case during 2018 as a result of the new regulations introduced by the ISSF [3] in response to the IOC’s call for gender equality [2]. The women’s average scores during the 2018 European Championship was always similar or higher than their 2016 European Championship scores. Therefore, these regulatory changes have achieved gender equality without detriment to the sporting excellence of women. In addition, after these modifications to equalize the number of shots given to men and women, neither male nor female performance was statistically different for rifle shooting, although the men’s pistol scores continued to be higher than the women’s average score.

These regulatory changes have already been analyzed by Goldschmied et al. [4], who did not find gender performance differences in the air rifle competition among US Collegiate competitors. Furthermore, Kemnitz et al. [20] found no differences in performance by gender in non-Olympic competitions or military shooting. Therefore, our results agree with those from previous studies. The only statistically significant difference we found in favor of men was in the rifle competition in 2016 when women fired 40 shots, but not in 2018 when they fired 60 shots. Moreover, this difference was only found in the sample group that participated in both European Championships. However, it is important to mention that past research did find that men performed better than women with 22-caliber rifles at 50 meters [24] and in military settings with rifles [8].

In the case of the pistol competition, all the comparisons in our study showed that men obtained better results than women, with large effect sizes (all *d* > 0.800), equating to performance differences equal to or higher than 0.900% in every case. These results agree with police pistol shooting studies, regardless of the caliber used [16,17], and also partially coincide with the study by Vučković et al. [21] who only found performance differences at the beginning, and not at the end, of the police pistol shooting training program. However, Mon [13] did not find differences in performance between men and women in the air pistol event when comparing the APS competition data for 60 versus 40 shots fired, respectively. Nevertheless, it is worth noting that this data was drawn from the National Championships which is a less competitive level than the European Championships in which sex-related differences may not be as clear.

Thus, in contrast to almost all other sports where men’s absolute performance is better than that of women, this present study provides evidence that there are no differences in performance between men and women in rifle shooting. In the case of pistol shooting, we did find performance differences of between 0.9% and 1.89% which are much smaller than for other sports such as athletics and swimming where the performance differences are around 10% [10,11]. One explanation for this smaller gender-related performance difference in pistol shooting compared to other sports may be that shooting requires less upper body strength [4]. In addition, although general physical abilities influence all sports performance [12], there is some consensus that other variables strongly affect shooting, including balance [13], specific strength [14], precision [15], and fine motor coordination [18]. Furthermore, it seems logical that the specific clothing worn for rifle shooting decreases the relevance of muscular strength in this sport and helps to stabilize and equalize men’s and women’s performances [25]. Regarding the pistol competition, it is possible that men’s performance is better because women may have greater difficulty in stabilizing the weapon as a result of strength differences, both in the shoulder abductor muscles [13,16] and in the finger flexor muscles, otherwise known as ‘grip strength’ [13,17]. It has also been suggested that balance plays a stronger role in rifle shooting than in pistol shooting [26].

In this study we did not see any evidence that prolonged exertion (extending 40 shooting attempts to 60) hurt women’s performance [8] because of increased fatigue [6]. In fact, in pistol, the group of women who participated in both championships performed better in 2018 when they fired 60 shots, and for the rifle competition, the women who participated in 2018 performed better than the women who participated in 2016. Although, this is the first study to analyze the effect that the pistol and rifle regulation change has had on shooting gender equality and performance, our results suggest that shooting sport is working in the right direction in the search of effective parity between men and women. In addition, it highlights the fact that coaches should understand the reasons for the differences in Olympic shooting performance, and prepare different training strategies for men and women on their pistol and rifle teams to help compensate for these differences.

On the other hand, in our study, the scores of the male medalists were superior to the women medalists in all the cases analyzed, both in pistol and in rifle. However, these data are different from those of the last world cup held in New Delhi in February 2019. In this competition, the scores of women medalists in rifle were superior to men results, obtaining even the world record in women with a score of 634 points which is higher than that of men with 633.5 points. In the case of pistol, the three men medalists had superior results to women [23]. This data suggest that it would be interesting to analyze the rank order and the gender placement, as previous studies have done [4], focusing not only on the general average shooting performance but also, on the average performance for every block of 10 attempts.

Nevertheless, our study has some limitations which should be mentioned. The data set is limited because we only analyzed two European championships, both in the same facilities; in this research context this can be interpreted both as a controlling factor and as a weakness. Thus, it is important to highlight that future longitudinal studies should incorporate a wider range of competitive levels, including world championships, world cups and non-elite shooters, to know exactly the differences in shooting performance by gender, competition event and its possible evolution over time.

## Conclusions

The modified regulations introduced by the ISSF to promote gender parity, equating the competition for women and men, was an appropriate policy change and is in tune with social demands [27]. It optimizes competition and performance [1], and as we have demonstrated in this research, women’s performance did not decrease with the increased number of shots, and in the case of the rifle competition, there were no differences in performance between women and men.

## Supporting information

S1 Dataset 16_&_18. SPSS statistics data document number 1.
(SAV)

S2 Dataset 16_VS_18. SPSS statistics data document number 2.
(SAV)
